# Enterovirus D68 infection causing plastic bronchitis and severe pneumonia: a case report and literature review

**DOI:** 10.3389/fmed.2026.1977512

**Published:** 2026-10-16

**Authors:** Chenxi Lin, Tingting Tian, Zhiyan Hao, Ying Huang, Weimin Tian

**Affiliations:** Department of Pediatrics, Pudong Hospital Affiliated to Fudan University, Shanghai, China

**Keywords:** case report, Enterovirus D68, plastic bronchitis, rhinovirus, severe pneumonia

## Abstract

**Background:**

Plastic bronchitis (PB) is a rare disease characterized by the formation of tree-like or tubular mucus plugs within the bronchi, which can lead to severe airway obstruction and respiratory failure, potentially becoming life-threatening if diagnosis and treatment are delayed. Enterovirus D68 (EV-D68) is a common pathogen that causes acute respiratory infections in children,which can lead to wheezing, acute flaccid myelitis, and even respiratory failure. Nevertheless, a direct association between EV-D68 infection and plastic bronchitis has not been previously reported. As the first documented case, this report establishes the association between EV-D68 and plastic bronchitis, thereby expanding the clinical spectrum of EV-D68.

**Methods:**

We report a case of plastic bronchitis and severe pneumonia caused by Enterovirus D68 infection. To identify additional cases and elucidate the clinical characteristics of this condition, a systematic literature search was conducted using PubMed, Embase, and Web of Science databases for the period from January 2006 to June 2026. The search strategy employed the following keywords: “Enterovirus D68” “plastic bronchitis” and “severe pneumonia”.

**Case description:**

This case involved an 11-year-old girl with previously good health. Chief complaints: cough and wheezing for 1 day, chest pain for half a day. Chest CT showed patchy increased-density opacities in the left upper loben with reduced left upper lobe volume, small nodular increased-density opacities in the left lower lobe and right lower lobe, right pleural thickening. Following admission, two flexible bronchoscopic examinations with plastic cast removal and bronchoalveolar lavage (BAL) were performed; bronchoscopic findings were consistent with plastic bronchitis and bronchial mucosal inflammation. Targeted next-generation sequencing (tNGS) of bronchoalveolar lavage fluid(BALF) detected Enterovirus D68 and Rhinovirus. Intravenous immunoglobulin support and methylprednisolone anti-inflammatory therapy were also administered. By hospital day 10, the patient's condition had stabilized;Follow-up chest CT demonstrated resolution of the left upper lobe patchy high-density opacities with re-expansion of the left upper lobe, patchy high-density opacities in the left lower lobe and small left pleural effusion. The newly developed lesion in the left lower lobe was considered resolving inflammatory changes rather than new active infection. The patient was discharged with medication. At 6 days post-discharge, follow-up chest CT demonstrated complete resolution of pulmonary infiltrates, pleural effusions, consistent with full clinical recovery. The total duration of treatment was 10 days of hospitalization plus 6 days of oral therapy after discharge, giving a total of 16 days.

**Conclusion:**

EV-D68 infection should be recognized as a potential etiology of plastic bronchitis or severe pneumonia in children. In addition to direct pathogen-mediated tissue injury, immune-mediated damage warrants careful consideration in the underlying pathophysiological mechanisms. Early bronchoscopic intervention combined with anti-inflammatory and immunomodulatory therapy may prove critical in shortening the disease course and improving clinical outcomes.

## Introduction

EV-D68 maintained a sporadic after its initial isolation in 1962 until the 2014 North American outbreak garnered global attention (1), marking a turning point in recognizing this pathogen as a cause of severe respiratory disease spectrum. Subsequent surveillance has revealed a consistent association between EV-D68 circulation and clusters of severe acute respiratory illness in children requiring intensive care. Notably, EV-D68 has emerged as a leading cause of acute flaccid myelitis (AFM), yet its potential to trigger severe lower airway disease with plastic cast formation remains underappreciated. The clinical significance of recognizing plastic bronchitis (PB) in the context of EV-D68 outbreaks is threefold: first, early bronchoscopic intervention is critical for cast removal and airway clearance, directly impacting survival; second, the inflammatory mechanisms underlying EV-D68-associated PB, particularly the role of immune-mediated injury and eosinophilic infiltration, may inform targeted therapeutic strategies including corticosteroids and immunoglobulin therapy; third, heightened awareness among clinicians during EV-D68 epidemic seasons may facilitate timely diagnosis and reduce delays in definitive management. PB is a rare clinical syndrome characterized pathologically by the intrabronchial formation of tree-like or tubular casts, resulting in airway obstruction and ventilatory dysfunction. Based on underlying pathophysiological mechanisms, PB is classified into two subtypes: the lymphatic type (associated with lymphatic circulatory impairment, with casts rich in lymphatic fluid constituents) and the eosinophilic type (associated with infection, allergy, and inflammation, with casts containing abundant eosinophils) (2). Definitive diagnosis of PB relies upon bronchoscopic retrieval of casts with subsequent histopathological or molecular subtyping; management necessitates precision intervention tailored to the specific subtype, with eosinophilic PB warranting anti-inflammatory, antimicrobial, and corticosteroid therapy (2). During the acute phase, bronchoscopic cast extraction remains the critical life-saving intervention. In the pediatric population, PB is most commonly associated with respiratory tract infections (e.g., Mycoplasma pneumoniae, viral, and bacterial pathogens), immune dysregulation, congenital airway malformations (e.g., primary ciliary dyskinesia, cystic fibrosis), postoperative congenital heart disease, lymphatic disorders, and asthma. Although respiratory tract infections have been established as the predominant etiology of pediatric plastic bronchitis, with an increasing body of literature in recent years, the association between EV-D68 and PB has not been directly reported globally and remains at the “case report—mechanistic hypothesis” stage, necessitating urgent validation through multicenter collaborative studies. Moreover, the optimal therapeutic strategy for viral infection–associated PB remains ill-defined, with an absence of standardized protocols for antiviral or immunomodulatory interventions.

## Case presentation

An 11-year-old girl with an unremarkable past medical history presented with chief complaints of cough and wheezing for 1 day and chest pain for half a day. No significant family history of disease. Upon admission, the patient exhibited deep, productive cough with thick, tenacious sputum that was difficult to expectorate, accompanied by chest pain, dyspnea, and rhinorrhea, without fever or other constitutional symptoms. On physical examination: temperature 36.5 °C, heart rate 120 beats/min, respiratory rate 30 breaths/min, peripheral oxygen saturation 90%–94%, blood pressure 100/70 mmHg, body weight 45 kg. Breath sounds were slightly diminished on the left compared to the right, and no rales were auscultated bilaterally;Muscle strength 5/5 in all extremities, normal muscle tone, normal tendon reflexes, intact cranial nerves, normal coordination;remainder of the physical examination was unremarkable. Outpatient chest radiography demonstrated infiltrates in the upper and lower fields of the left lung(Figure 1A). Admission chest CT: patchy increased-density opacities in the left upper lobe with reduced left upper lobe volume, small nodular increased-density opacities in the left lower lobe and right lower lobe, right pleural thickening(Figures 2E–G). Echocardiography and electrocardiogram were unremarkable. Complete blood count, C-reactive protein, procalcitonin, coagulation studies, cardiac biomarkers, and hepatic transaminases were all within normal reference ranges(Table 1). Throat swab Mycoplasma pneumoniae nucleic acid was negative. Arterial blood gas analysis (room air) was consistent with type II respiratory failure and respiratory acidosis combined with metabolic acidosis (Table 2).

**Figure 1:**
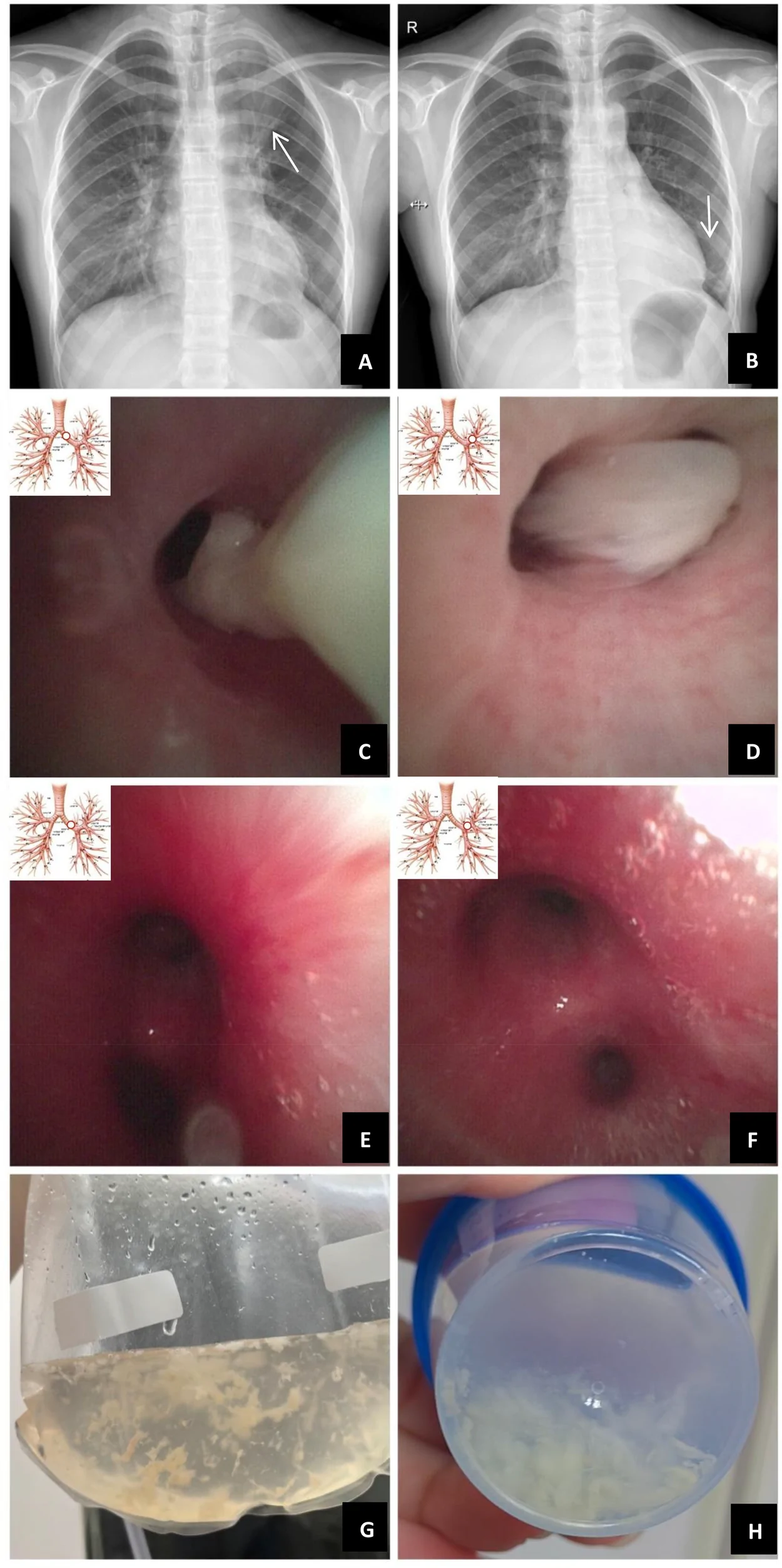
**(A)** chest radiograph obtained in the outpatient clinic on the day of admission: inflammation in the left upper and lower lung fields(→ labeled). **(B)** Repeat chest radiograph on hospital day 4 (post-bronchoscopy day 2): resolution of high-density opacities in the left upper lung field, with increased inflammation in the left lower lobe compared to prior imaging A(→ labeled). **(C,D,E,F,G)**. Flexible bronchoscopy performed on hospital day 2: **(C)** plastic sputum cast in the left main bronchus; **(D)** subsegmental sputum cast in the left upper lobe. **(E)** The openings of the left upper and lower lobe bronchi exposed after complete removal of the sputum cast from the left main bronchus, with hyperaemic mucosa. **(F)** The opening of the left upper lobe bronchus exposed after cast removal, with hyperaemic mucosa. **(G)** copious sputum casts visible in the lavage fluid. **(H)** Flexible bronchoscopy performed on hospital day 6:strip-like sputum casts visible in the lavage fluid.

**Figure 2:**
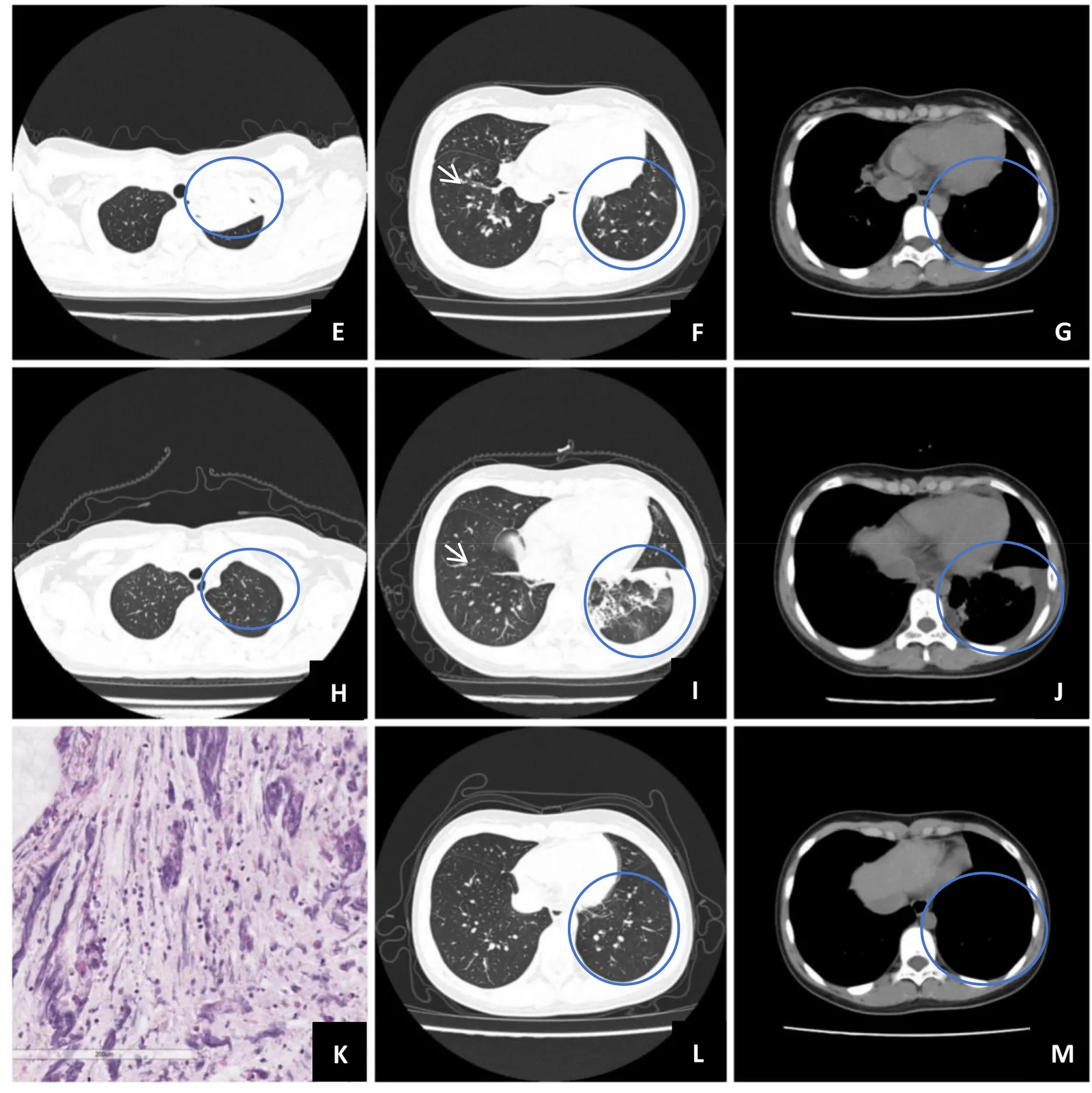
**(E,F,G)**. Chest CT performed on the day of admission: **(E)** patchy high-density opacities in the left upper lobe with reduced left upper lobe volume(○ labeled); **(F)** small nodule increased-density opacities in the left lower lobe and the right lower lobe. **(H,I,J)**. Chest CT on hospital day 10: **(H)** compared with **(E)**, demonstrating resolution of left upper lobe high-density opacities with re-expansion(○ labeled); **(I)** compared with **(F)**, showing resolution of right lower lobe inflammation(→ labeled) and newly developed inflammatory lesions in the left lower lobe(○ labeled); **(J)** compared with **(G)**, showing newly developed left-sided pleural effusion(○ labeled). **(K)**. Histopathological examination of the plastic sputum cast from C with hematoxylin-eosin (HE) staining: necrotic debris containing histiocytes, lymphocytes, eosinophils, and abundant neutrophils. **(L,M)**. Chest CT on treatment day 16: compared with **(I)** and **(J)**, showing resolution of pulmonary inflammation and pleural effusions(○ labeled).

**Table 1:** Complete blood count, inflammatory markers, lactate dehydrogenase, D-dimer.

Date	WBC ×10^9/L	NE ×10^9/L	LYM ×10^9/L	PLT ×10^9/L	CRP mg/L	PCT ng/mL	LDH U/L	DD mg/L
Day1	9.23	7.20	1.16	213	<0.50	<0.05	174	≤0.25
Day4	9.81	8.59	0.95	256	< 0.50	0.11	138	
Day10	11.14	6.62	3.42	251	3.46	0.16	140	

**Table 2:** Arterial blood gas analysis, chloride ion.

Date	pH	pCO2 KPa	pO2 KPa	SPO2%	HCO3- mmol/L	AG mmol/L	BE mmol/L	LAC mmol/L	CL- mmol/L
Day1	7.28	7.13	5.01	61.2	21.1	11.7	−1.9	2.9	108
Day10	7.50	4.11	13.5	99.2	25.8	9.0	0.8	2.0	105

Following admission, a comprehensive diagnostic workup was completed. The patient was placed under continuous oxygen saturation and cardiac monitoring, with supplemental oxygen delivered via nasal cannula. Intravenous ambroxol was administered as mucolytic therapy and intravenous doxycycline was initiated for empirical antimicrobial coverage（prior to pathogen identification, as the patient had no elevation in white blood cell count, CRP, PCT, or other inflammatory markers; considering that Mycoplasma pneumoniae infection is more common in older children and tends to cause large pulmonary consolidations or plastic bronchitis, and that macrolide-resistant Mycoplasma pneumoniae has become prevalent in China in recent years, empirical doxycycline was administered）. On hospital day 2, flexible bronchoscopic examinations with plastic cast removal and bronchoalveolar lavage (BAL) was performed. Bronchoscopic examination revealed copious yellow-white viscous secretions within the lumen of the right intermediate bronchus and medial basal segmental bronchus of the right lower lobe;a yellow-white strip-like sputum cast was observed obstructing the left main bronchus lumen (incomplete obstruction); strip-like sputum casts were seen obstructing the left upper lobe bronchial opening and subsegmental bronchial lumens (near-complete obstruction). After suction and clearance of sputum casts followed by bronchoalveolar lavage, the lumens of all segmental bronchi were patent; Diffuse hyperaemia of the bronchial mucosa was observed in all segments and subsegments of both lungs, without erosion, bleeding, or pseudomembranes;copious sputum casts visible in the lavage fluid (Figures 1C–G). The Bronchoscopic diagnosis was plastic bronchitis. Targeted next-generation sequencing (tNGS) of BALF identified Enterovirus D68 and Rhinovirus (Table 3). TNGS based on ultra-multiplex targeted amplification and high-throughput sequencing technology, detecting 101 bacteria, 55 fungi, 87 viruses, 6 parasites, 16 mycobacteria, and 15 mycoplasmas/chlamydiae/spirochetes/rickettsiae. Routine bacterial cultures of BALF and blood samples were both negative. Histopathological examination of the plastic sputum casts demonstrated necrotic debris with histiocytes, lymphocytes, eosinophils, and abundant neutrophils (Figure 2K). Intravenous immunoglobulin (IVIG) was administered for 2 days at a total dose of 1.5 g/kg. On hospital day 4, the patient remained dependent on nasal cannula oxygen and exhibited productive cough with yellow sputum; fine moist rales were auscultated bilaterally, and repeat chest radiograph demonstrated resolution of the left upper lobe high-density opacities,with increased inflammation in the left lower lobe compared to prior imaging(Figure 1B). Given that the chest CT the day before bronchoscopy had predominantly shown left upper lobe inflammation with atelectasis, with only minimal inflammatory exudation in the right lower lobe, yet the first bronchoscopy revealed copious secretions pouring from the right intermediate bronchus and lower lobe bronchus, this indicated rapid disease progression. Combined with the patient's persistent symptoms and signs, this further validated ongoing bilateral pulmonary inflammation with progressive trend, thus meeting indications for a second bronchoscopic intervention. On hospital day 6, a second flexible bronchoscopy with bronchoalveolar lavage was performed; Bronchoscopic examination revealed hyperaemic bronchial mucosa in all segments and subsegments of both lungs; Moderate secretions and sputum casts obstructing the orifices of the right lower lobe superior segment and right middle lobe bronchi, as well as abundant gray-white viscous secretions pouring from the orifices of the left lower lobe superior segment and lateral basal segment bronchi;All secretions and sputum casts were suctioned and cleared, with strip-like sputum casts visible in the lavage fluid (Figure 1H). Meanwhile, doxycycline was discontinued and replaced with intravenous ceftriaxone for anti-infective treatment(The patient's throat swab Mycoplasma pneumoniae nucleic acid was negative, and Mycoplasma pneumoniae was not detected in the BALF tNGS results, excluding Mycoplasma pneumoniae infection and confirming EV-D68 and rhinovirus infection; however, after the first bronchoscopic bronchoalveolar lavage, the patient gradually developed productive cough with yellow sputum, with persistent bilateral moist rales, and had undergone two invasive procedures [bronchoscopic bronchoalveolar lavage], making bacterial co-infection impossible to exclude, hence antibiotic adjustment). During the course of illness, oxygen saturation monitored under nasal cannula oxygen therapy remained within the normal range. By hospital day 8, the patient's respiratory status had stabilized, oxygen saturation normalized, and pulmonary crackles diminished; oxygen therapy was discontinued. On hospital day 10, the patient's condition had stabilized; follow-up chest CT demonstrated resolution of the left upper lobe patchy high-density opacities with re-expansion of the left upper lobe,patchy high-density opacities in the left lower lobe, small left pleural effusions (Figures 2H–J). The newly developed lesion in the left lower lobe was considered resolving inflammatory changes rather than new active infection, based on the following: (1) overall clinical improvement with stabilized vital signs; (2) reduced oxygen requirements with normal oxygen saturation; (3) alleviation of respiratory symptoms with gradually decreasing pulmonary rales; (4) chest radiograph, two bronchoscopic findings, and daily pulmonary signs indicated that the patient initially presented with predominantly left upper lobe inflammation, progressing to bilateral lower lobe inflammation. The patient met discharge criteria: clinical stability, ability to tolerate oral medications, with follow-up scheduled to monitor residual changes. No neurological deficits were identified during hospitalization, and no limb weakness or cranial nerve palsies developed. The patient was ambulatory at discharge. Discharge was granted with medications. Discharge diagnosis: severe pneumonia (Enterovirus D68 and Rhinovirus infection), plastic bronchitis, type II respiratory failure, respiratory acidosis combined with metabolic acidosis, pleural effusion.

**Table 3:** BALF tNGS positive results(病毒列表 **virus list,**类型type,病原名称pathogen name,序列数sequence count,病原估算浓度estimated pathogen concentration).

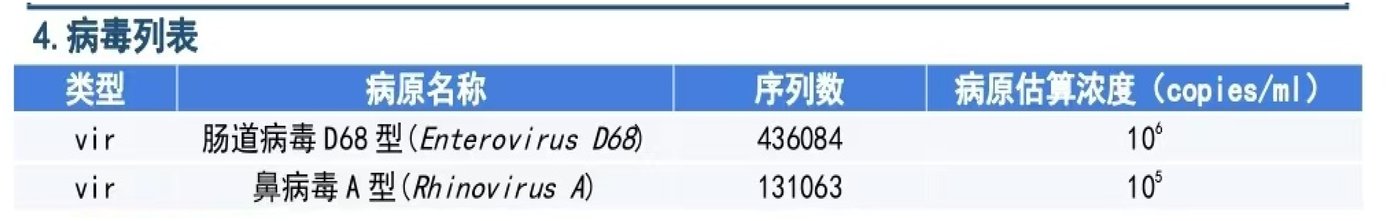

At 6 days post-discharge, the patient remained asymptomatic with no abnormal physical findings, including normal neurological examination; follow-up chest CT demonstrated complete resolution of pulmonary infiltrates and pleural effusions(Figures 2L,M). Pharmacological management during hospitalization included (Timeline of the current treatment course, Figure 3): antimicrobial therapy (intravenous doxycycline 2 mg/kg,q12h,no loading dose,for 5 days, intravenous ceftriaxone 1 g,qd, for 5 days), intravenous ambroxol, human intravenous immunoglobulin (IVIG) at a cumulative dose of 1.5 g/kg administered over 2 days (1 g/kg + 0.5 g/kg), intravenous methylprednisolone for 10 days (40 mg every 12 h for 4 days, 30 mg every 12 h for 3 days, 30 mg once daily for 2 days, 20 mg once daily for 1 day), nebulized budesonide plus inhaled acetylcysteine, and oral calcitriol and calcium carbonate to prevent osteoporosis from prolonged corticosteroid-induced calcium loss. Post-discharge medications included: oral methylprednisolone with tapering every 2 days (16mg–12mg–8 mg, once daily), and nebulized budesonide plus inhaled acetylcysteine twice daily. The entire diagnosis and treatment course was relatively smooth, and both the family and the patient were satisfied. However, there remain points for consideration: the choice of antibiotics—whether mixed bacterial infection was possible when the child had yellow sputum with persistent bilateral lung rales, and whether antibiotics could have been avoided.

**Figure 3:**
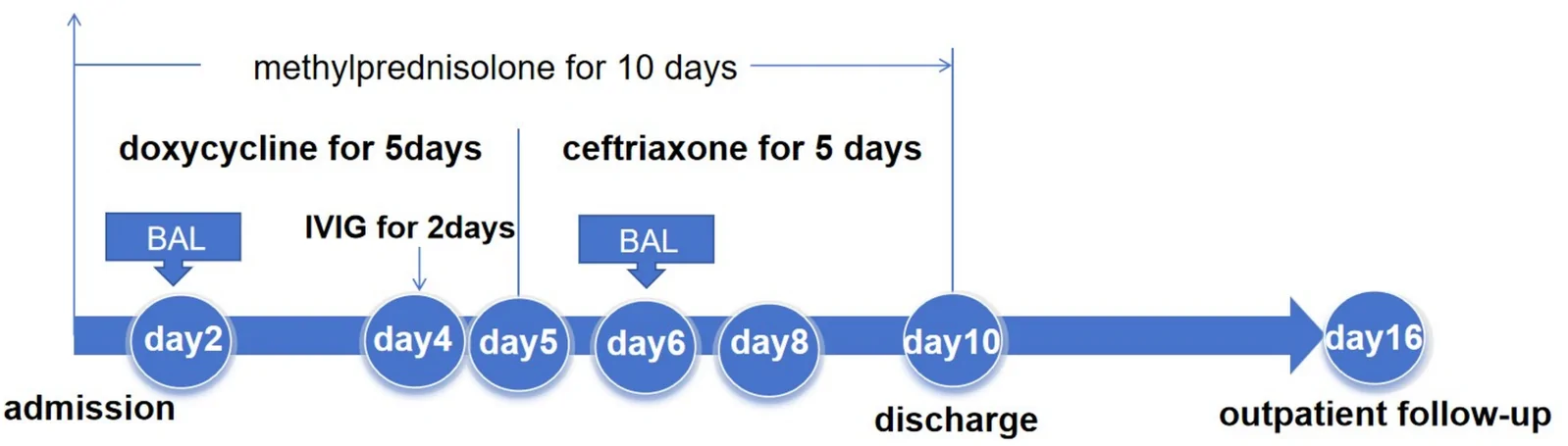
Timeline of the current treatment course.

This patient presented with acute onset and rapid clinical progression,with definitive diagnosis by chest imaging, BALF tNGS confirming Enterovirus D68 and Rhinovirus as the causative pathogens. A total of two flexible bronchoscopy procedures with plastic cast removal and BAL were performed, in conjunction with intravenous immunoglobulin supportive therapy and methylprednisolone anti-inflammatory treatment. The entire therapeutic course spanned 16 days, with 10 days of hospitalization and 6 days of oral medications post-discharge,achieving of complete clinical recovery.

## Literature review

### Search strategy

We report a case of plastic bronchitis and severe pneumonia caused by Enterovirus D68 infection. To identify additional cases and elucidate the clinical characteristics and underlying mechanisms of this condition, a systematic literature search was conducted using PubMed, Embase, and Web of Science databases for publications from January 2006 to June 2026 regarding Enterovirus D68 infection associated with plastic bronchitis or severe pneumonia in children. The search strategy employed the following keywords: (“Enterovirus D68” OR “EV-D68”) AND (“Severe Pneumonia” OR “Plastic Bronchitis” OR “Bronchial Cast” OR “Mucus plugs” OR “Molded Sputum Plug” OR “Plastic Expectoration” OR “Respiratory Failure”).

### Inclusion and exclusion criteria

Inclusion and exclusion criteria were as follows: Inclusion criteria for literature search (2006–2026): (a) Confirmed respiratory tract infection with documented EV-D68 infection; (b) Clinical diagnosis of severe pneumonia or plastic bronchitis;(c) Diagnosis of EV-D68-associated severe pneumonia based on established guidelines for pediatric community-acquired pneumonia, corroborated by microbiological testing; (d) Age ≤ 18 years.

### Data extraction and analysis

A total of 156 relevant articles were retrieved, of which 28 directly described EV-D68-associated severe pneumonia. and none mentioned therapeutic bronchoscopic airway management (plastic bronchitis or cast removal). Although the search was systematic and comprehensive, no previously confirmed EV-D68 plastic bronchitis cases were identified in our review, with no pathological description of tree-like casts under bronchoscopy in EV-D68 infection, and no direct evidence from EV-D68-associated PB animal models.

### Treatment and prognosis

Currently, no specific antiviral agents are available; management is primarily supportive and symptomatic: (a) Oxygen therapy and respiratory support, with mechanical ventilation required for severe cases; (b) Early bronchoscopic intervention for mucus cast removal, which is critical for PB; (c) Glucocorticoid administration remains controversial and may be indicated in patients with significant airway hyperresponsiveness; (d) Human intravenous immunoglobulin (IVIG): has become standard therapy, likely exerting its effects through passive immunization to limit viral infection progression or enhance viral clearance, in addition to its anti-inflammatory properties (3, 4). In an EV-D68 acute flaccid myelitis (AFM) mouse model, prophylactic IVIG administration demonstrated protective effects against paralysis; however, this was limited to early-stage infection (3, 4). The prognosis for most EV-D68 respiratory infections is favorable (1).

## Discussion

The case, confirmed by flexible bronchoscopy procedures with plastic cast removal and BAL were performed and BALF tNGS with detailed therapeutic documentation, provides valuable insights into the rare association between EV-D68 and plastic bronchitis in older children. This case reveals three key findings: First, EV-D68 can induce plastic bronchitis in previously healthy school-aged children,causing airway obstruction leading to respiratory distress and even respiratory failure, thereby expanding the known clinical spectrum of this infection. Evidence of airway obstruction: In this patient, chest imaging demonstrated atelectasis of the left upper lobe; bronchoscopy revealed complete or incomplete obstruction of the left main bronchus and left upper lobe bronchus lumen by plastic sputum casts; chest radiograph performed 2 days after clearance of intrabronchial casts showed re-expansion of the left upper lobe; arterial blood gas analysis indicated respiratory acidosis (elevated PaCO₂ due to airway obstruction and hypoventilation) combined with metabolic acidosis (elevated lactate due to tissue hypoxia). All of the above support our argument of airway obstruction. Based on the histopathological findings in this patient, infection-related PB was considered. Second, for patients with rapidly progressive disease accompanied by hypoxemia and radiological deterioration, early bronchoscopic examination is warranted; the removal of plastic secretions and tNGS-based pathogen identification are critical for diagnosis and targeted therapy. Third, complete recovery was achieved after 16 days of comprehensive treatment incorporating repeated bronchoscopic lavage combined with intravenous immunoglobulin and methylprednisolone, suggesting that aggressive multimodal intervention may be pivotal for favorable outcomes in EV-D68–associated severe PB.

In this case, histopathological examination of the plastic sputum cast revealed necrotic debris containing histiocytes, lymphocytes, eosinophils, and abundant neutrophils. Pathophysiological Mechanism of Infection-Related PB Evolution:Infection → Inflammation → Mucus Hypersecretion → Sputum Plug Formation → Fibrin Exudation → Cellular Infiltration → Cast Solidification → PB.Plastic sputum casts refer to solidified sputum plugs that form *in situ* within the bronchial tree, exhibiting morphological characteristics of bronchial branching architecture with dense, elastic consistency. The pathological essence represents the advanced solidified form of a mucus-inflammatory-cellular debris complex, constituting the core pathological entity of PB. Literature has demonstrated that extremely concentrated mucins (MUC5AC/MUC5B) can form gel-like solids through disulfide cross-linking without requiring a fibrin scaffold. Therefore, the currently broadly defined plastic bronchitis can be diagnosed based on the triad of “tree-like structure+mucus solidification+inflammatory cell infiltration,” with fibrin serving as supportive rather than obligatory evidence. Based on these findings, we hypothesize that EV-D68 may contribute to PB formation through the following three interconnected mechanisms（Figure 4):(1) Direct epithelial injury and impairment of mucociliary clearance: EV-D68 invades bronchial ciliated epithelium via sialic acid receptors,disrupting mucociliary function and causing mucus stasis (5–7). (2) Mucus hypersecretion: Studies have found that the EV-D68 VP3 protein interacts with mitochondrial antiviral signaling protein (MAVS) to modulate NF-*κ*B signaling:early inhibition facilitates immune evasion,while late viral replication triggers NF-*κ*B overactivation,upregulating MUC5AC transcription and promoting viscous mucus production that impairs mucus transport (8, 9).(3) Inflammatory cascade and cast formation: Existing studies have found that EV-D68 induces neutrophil extracellular traps (NETs) through IL-17-mediated NADPH oxidase-dependent NETosis;released DNA, histones,and elastase increase mucus elasticity. Activated eosinophils degranulate cytotoxic proteins (MBP, EPX), damaging epithelium and promoting mucus plug solidification (10–14). These pathways converge to form the characteristic plastic casts of PB.

**Figure 4:**
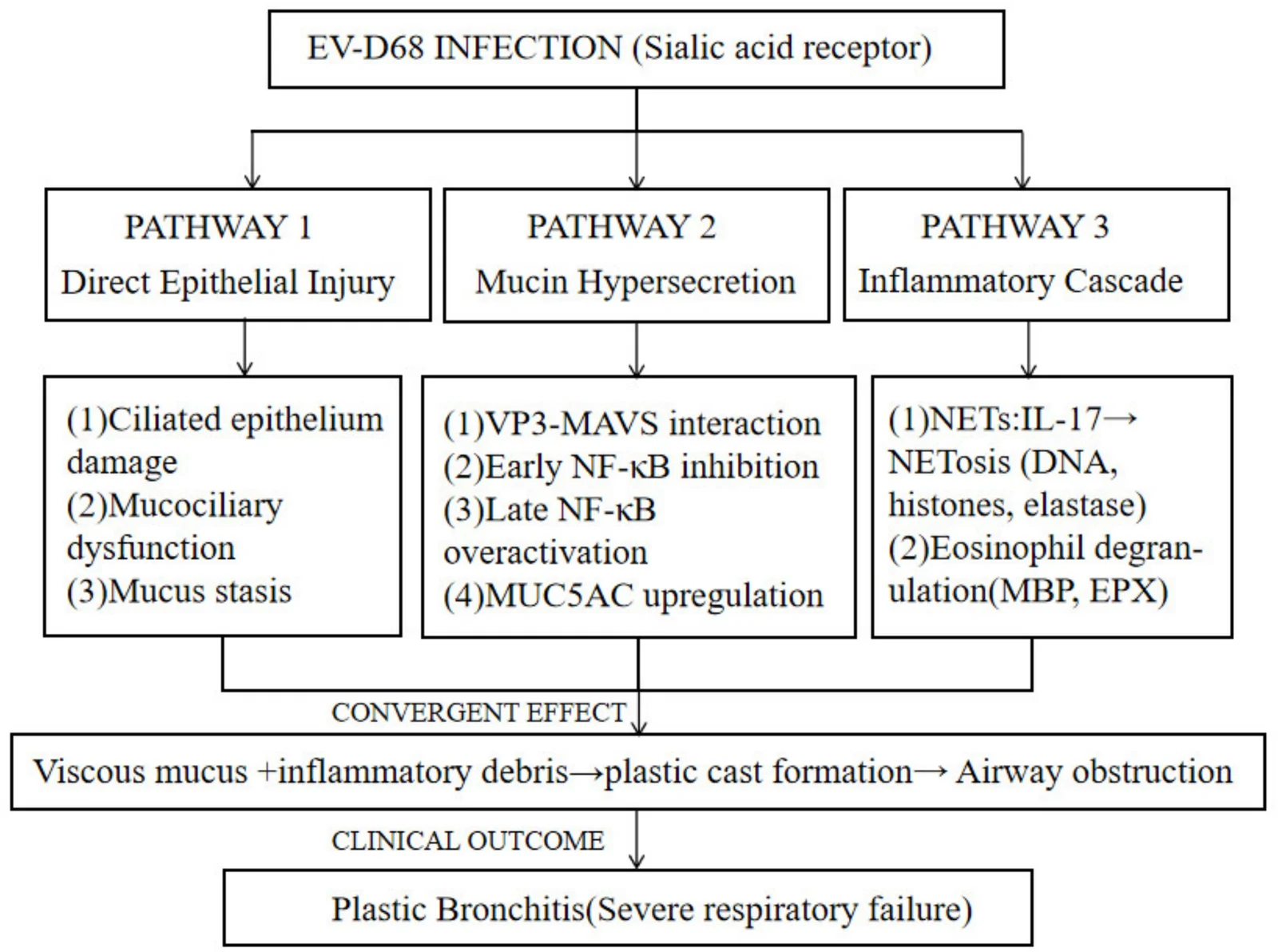
Proposed mechanisms of EV-D68-induced plastic bronchitis.

EV-D68 infection is frequently complicated by co-infection with other pathogens; synergistic inflammatory responses may promote PB development. During the 2024 outbreak in Italy, 40% of cases exhibited co-infection, while a 2014–2018 survey in Guangdong, China, found that 72.7% of cases had mixed infections, predominantly with rhinovirus, respiratory syncytial virus and bacteria (15, 16)**.** This case identified co-infection with EV-D68 and rhinovirus. The underlying mechanisms for this co-occurrence are analyzed as follows: (a) Phylogenetic relatedness and shared tropism (17, 18): Both viruses belong to the Picornaviridae family. Moreover, EV-D68 exhibits acid lability and low-temperature growth characteristics similar to rhinovirus. Both are primarily transmitted via respiratory droplets and demonstrate particular tropism for respiratory epithelial cells, creating spatial convenience for mixed infection. (b) Hijacking of shared cellular pathways (19): Studies have demonstrated that both viruses actively hijack the CXCL8/MAPK/hnRNP-K signaling axis during host cell infection. Through this mechanism, viruses recruit hnRNP-K protein, which is essential for replication, from the nucleus to the cytoplasm to facilitate viral proliferation. The shared utilization of this pathway implies that once one pathogen establishes infection and activates this axis, the replication efficiency of the other is consequently enhanced. (c)Overlapping epidemic seasons (20): Both viruses predominantly circulate during summer and autumn months (August through November in the Northern Hemisphere) and represent common pathogens causing acute respiratory infections in children, substantially increasing the probability of simultaneous encounter within the same host during the same temporal window. The BALF tNGS of this patient detected both EV-D68 and rhinovirus. However, the read count of EV-D68 was significantly higher than that of rhinovirus (436,084 vs. 131,063, see Table 3). Furthermore, the patient's clinical presentation (severe pneumonia) was consistent with the known severe disease spectrum of EV-D68, rather than typical rhinovirus infection. Therefore, EV-D68 was considered the most likely primary pathogen in this patient, although rhinovirus co-infection cannot be completely excluded as a contributing factor to disease exacerbation and progression.

In conclusion, this case represents the first documented report of plastic bronchitis associated with Enterovirus D68 infection in a previously healthy child, expanding the clinical spectrum of severe EV-D68 disease. The successful management through early bronchoscopic intervention combined with immunomodulatory therapy highlights the importance of timely recognition and multidisciplinary treatment. However, given the inherent limitations of a single-case observation, our findings require validation through multicenter collaborative studies with larger cohorts to establish the true incidence and risk factors of EV-D68-associated plastic bronchitis. Furthermore,In light of this case, the following conclusions can be drawn:For patients presenting with sudden onset, short disease duration, rapid progression, and predominant dyspnea, with exclusion of acute laryngeal obstruction, acute asthma exacerbation, airway foreign body, pulmonary embolism, and pneumothorax as alternative etiologies of respiratory distress, a high index of clinical suspicion for plastic bronchitis or extensive airway mucus plugging resulting in severe pneumonia is warranted. Chest CT imaging holds significant value in disease assessment, whereas bronchoscopic examination and intervention remain irreplaceable in both diagnosis and management. Prompt identification of the causative pathogen is paramount for guiding therapeutic strategies and prognostic evaluation. Extensive pulmonary consolidation is not exclusive to Mycoplasma and bacterial infections; viral etiologies must not be overlooked. This case demonstrates that EV-D68 infection should be recognized as a potential etiology of plastic bronchitis and severe pneumonia in previously healthy school-aged children. The successful multimodal treatment in this case underscores the necessity of prospective studies to establish standardized protocols for combined bronchoscopic lavage, immunoglobulin, and glucocorticoid therapy. Furthermore, enhanced EV-D68 surveillance and reporting systems are warranted to better delineate its clinical spectrum and inform public health responses to severe respiratory outbreaks. The association between EV-D68 and PB remains at the “case report—mechanistic hypothesis” stage, urgently requiring validation through multicenter collaborative studies. EV-D68-associated AFM: this patient did not develop this complication, but clinicians should maintain vigilance for neurological symptoms in severe EV-D68 infections. Future directions: (1) Establishment of standardized bronchoscopic assessment protocols for severe EV-D68 cases; (2) Exploration of molecular markers for viral clade–specific PB susceptibility; (3) Development of broad-spectrum enterovirus vaccines for prevention of severe complications. Clinicians should heighten awareness of the diverse clinical manifestations of EV-D68 infection, maintaining particular vigilance for rare complications such as PB in healthy children or those with underlying conditions including asthma.

## Data Availability

The original contributions presented in the study are included in the article/Supplementary Material, further inquiries can be directed to the corresponding authors.

## References

[1] Midgley CM Watson JT Nix WA Curns AT Rogers SL Brown BA. Severe respiratory illness associated with a nationwide outbreak of enterovirus D68 in the USA (2014): a descriptive epidemiological investigation. Lancet Respir Med. (2015) 3(11):879–87. 10.1016/S2213-2600(15)00335-5 26482320 PMC5693332

[2] Wang H Yu X Lin Q Meng F Li Y. Plastic bronchitis: a narrative review of the classification methods and pathogenesis. Front Pediatr. (2026) 14:1814321. 10.3389/fped.2026.1814321 42212108 PMC13212312

[3] Elrick MJ. Enterovirus D68 molecular and cellular biology and pathogenesis. J Biol Chem. (2021) 296:100317. 10.1016/j.jbc.2021.100317 33484714 PMC7949111

[4] Hixon AM Clarke P Tyler KL. Evaluating treatment efficacy in a mouse model of enterovirus D68–associated paralytic myelitis. J Infect Dis. (2017) 216(10):1245–53. 10.1093/infdis/jix468 28968718 PMC5853295

[5] Liu Y Sheng J Baggen J Meng G Xiao C Thibaut HJ. Sialic acid-dependent cell entry of human enterovirus D68. Nat Commun. (2015) 6(1):8865. 10.1038/ncomms9865 26563423 PMC4660200

[6] Liu Y Sheng J Fokine A Meng G Shin W-H Long F. Structure and inhibition of EV-D68, a virus that causes respiratory illness in children. Science. (2015) 347(6217):71–4. 10.1126/science.1261962 25554786 PMC4307789

[7] Sooksawasdi Na Ayudhya S Laksono BM Van Riel D. The pathogenesis and virulence of enterovirus-D68 infection. Virulence. (2021) 12(1):2060–72. 10.1080/21505594.2021.1960106 34410208 PMC8381846

[8] Chen H Huang M Hou B Liu Z Tan R Cui L. The structural protein VP3 of enterovirus D68 interacts with MAVS to inhibit the NF-*κ*B signaling pathway. J Virol. (2025) 99(4):1–17. 10.1128/jvi.00163-25 PMC11998529 40042308

[9] Bonser LR Zlock L Finkbeiner W Erle DJ. Epithelial tethering of MUC5AC-rich mucus impairs mucociliary transport in asthma. J Clin Invest. (2016) 126(6):2367–71. 10.1172/JCI84910 27183390 PMC4887179

[10] Rajput C Han M Bentley JK Lei J Ishikawa T Wu Q. Enterovirus D68 infection induces IL-17-dependent neutrophilic airway inflammation and hyperresponsiveness. JCI Insight. (2018) 3(16):e121882. 10.1172/jci.insight.121882 30135310 PMC6141171

[11] Grizer CS Elliott KC Singh G Vogt MR Mattapallil JJ. Respiratory infection with enterovirus D68 induces severe acute lung inflammation in the pediatric ferret model. iScience. (025) 28(8):113071. 10.1016/j.isci.2025.113071 PMC12302187 40727933

[12] Qian F Qiang L Chanli L Caiyuan S Chan X Mei C. Mucus plugs: a core pathophysiological mechanism in COPD and a potential imaging biomarker of disease activity. Egypt J Bronchol. (2026) 20(1):1–14. 10.1186/s43168-026-00603-6

[13] Linssen RS Chai G Ma J Kummarapurugu AB Van Woensel JBM Bem RA. Neutrophil extracellular traps increase airway mucus viscoelasticity and slow mucus particle transit. Am J Respir Cell Mol Biol. (2021) 64(1):69–78. 10.1165/rcmb.2020-0168OC 33095650 PMC7780998

[14] Steffan BN Townsend EA Denlinger LC Johansson MW. Eosinophil-epithelial cell interactions in asthma. Int Arch Allergy Appl Immunol. (2024) 185(11):1–15. 10.1159/000539309 PMC11534548 38885626

[15] Tang S-H Yuan Y Xie Z-H Chen M-J Fan X-D Guo Y-H. Enterovirus D68 in hospitalized children with respiratory symptoms in guangdong from 2014 to 2018: molecular epidemiology and clinical characteristics. J Clin Virol. (2021) 141:104880. 10.1016/j.jcv.2021.104880 34153861

[16] Messali S Bertelli A Dotta L Giovanetti M Sclavi L Venneri G. Outbreak of enterovirus D68 in young children, brescia, Italy, August to November 2024. J Med Virol. (2025) 97:e70372. 10.1002/jmv.70372 40297997 PMC12038777

[17] Rahajamanana VL Thériault M Rabezanahary H Sahnoun YG Mallet MC Isabel S. Advances in the treatment of enterovirus-D68 and rhinovirus respiratory infections. Infect Dis Rep. (025) 17(61):1–22. 10.3390/idr17030061 PMC12193398 40559192

[18] Gui Y Han B Wei J Sun D Zhou L Yuan S. Respiratory enterovirus D68: virology, clinical surveillance, host-pathogen interactions, and therapeutic prospects. Front Cell Infect Microbiol. (2026) 16:1774711. 10.3389/fcimb.2026.1774711 41868141 PMC12999577

[19] Yang Q Guo H Li H Li Z Ni F Wen Z. The CXCL8/MAPK/hnRNP-K axis enables susceptibility to infection by EV-D68, rhinovirus, and influenza virus *in vitro*. Nat Commun. (2025) 16:1715. 10.1038/s41467-025-57094-0 39962077 PMC11832783

[20] Clopper BR Lopez AS Goldstein LA Ng TFF Toepfer AP Staat MA. Enterovirus D68-associated respiratory illness in children. JAMA Netw. Open. (2025) 8(5):e259131. 10.1001/jamanetworkopen.2025.9131 40338550 PMC12062906

